# Influence of Nd:YAG laser on the penetration of a bioceramic root canal sealer into dentinal tubules: A confocal analysis

**DOI:** 10.1371/journal.pone.0202295

**Published:** 2018-08-22

**Authors:** Rodrigo Jardim Del Monaco, Marcelo Tavares de Oliveira, Adriano Fonseca de Lima, Ricardo Scarparo Navarro, Raquel Virgínia Zanetti, Daniela de Fátima Teixeira da Silva, Anna Carolina Ratto Tempestini Horliana

**Affiliations:** 1 Postgraduate Program in Biophotonics Applied to Health Sciences, University Nove de Julho, São Paulo, SP, Brazil; 2 Department of Dentistry, Ibirapuera University, São Paulo, SP, Brazil; 3 Department of Dental Research Division, Paulista University, São Paulo, SP, Brazil; 4 Department of Postgraduate Program in Bioengineering and Biomedical Engineering, University Brazil, São Paulo, SP, Brazil; 5 Postgraduate Program in Prosthetic Dentistry, São Leopoldo Mandic University, Campinas, SP, Brazil; Institute of Materials Science, GERMANY

## Abstract

**Objective:**

The aim of this *in vitro* study, is to evaluate the penetration of a bioceramic root canal sealer into dentinal tubules at 3 mm and 5 mm from the apex after Nd:YAG laser irradiation.

**Methods:**

Forty freshly extracted human mandibular premolars were prepared using Reciproc^®^ and irrigated with 17% ethylenediaminetetraacetic acid (EDTA). Teeth were divided into 4 groups: group 1, obturated with control sealer (AH Plus^®^); group 2, obturated with bioceramic sealer (Endosequence BC Sealer^®^); group 3, Nd:YAG laser + control sealer (AH Plus^®^); and group 4, Nd:YAG laser + bioceramic sealer (Endosequence BC Sealer^®^). The samples were transversely sectioned 3 mm and 5 mm from the apex and examined using confocal laser scanning microscopy. Two parameters were measured: 1) sealer penetration into dentinal tubules of the root canal and 2) sealer penetration into the perimeter of the root canal walls.

**Results:**

Penetration analysis showed that bioceramic sealer had a higher penetration at depths of 3 and 5 mm than that of the control sealer, regardless of laser use (p <0.05). Perimeter analysis showed that there was no difference between both sealers at a depth of 3 mm (p <0.05), regardless of laser use. At a depth of 5 mm, bioceramic sealer and laser showed a greater perimeter of penetration (p <0.05) than the control sealer.

**Conclusion:**

The use of Nd:YAG laser did not compromise the penetration of bioceramic sealer into dentinal tubules of root canals at 3 mm and 5 mm from the apex.

## Introduction

Mechanization of root canal instrumentation has made endodontic treatment more precise, efficient, and fast [1]. As a result, the time for which the irrigant stays in contact with the surface of a root canal has decreased; this has made these substances less efficient because they are time-dependent [2,3]. Instrumentation alone cannot completely decontaminate the root canals [4–6], making auxiliary therapies important. Several auxiliary techniques, such as high power lasers, photodynamic therapy, new instruments, and new irrigation approaches have been used to decontaminate dentinal tubules [5,7–15]. Currently, high power lasers are used in endodontic treatment. Particularly, Nd:YAG laser is known to have antimicrobial action and seals dentinal tubules [8,9,12,15,16]. Nd:YAG lasers can promote vaporization in the presence of a smear layer, causing fusion/resolidification of the dentin surface, which may decrease permeability, prevent reinfection, and reduce apical infiltration [17–20]. Despite its importance in cleaning and disinfection, the sealing of dentinal tubules could be a disadvantage because it may interfere with the penetration of sealers into root canals [19–22].

Recently, bioactive and osteogenic materials have been developed successfully in dentistry [23–26]. In endodontics, bioceramic sealers promotes dentine re-mineralization, have acceptable cytotoxicity, shows an antibacterial effect and good dentinal tubule penetration [25–28]. It was demonstrated that bioceramic sealers promotes penetration into dentinal tubules at 2 mm from apex with different obturation techniques [29]. Also, a retrospective study shows that obturations with bioceramic sealers could achieve 90,9% of success rate after 30.1 months follow up [27]. Bioceramic sealer was developed as a permanent root canal filling [27,28]. It does not shrink during setting, which increases its sealing capacity [23]. In addition, it has the ability to adhere and chemically bond to the root canal wall [30]. This mechanical locking is important as it decreases leakage [31,32]; however the obliteration of dentinal tubules by Nd:YAG lasers could be a disadvantage when this sealer is used. It could not penetrate into dentinal tubules, losing its benefits, since bioceramic sealers were developed to interact with non-irradiated dentin [33–38].

To date, only one study analyzed the interaction of bioceramic sealer with different dentin treatments, but in this study an Er:YAG laser was used [39]. Thus, the interaction of bioceramic sealers with dentin surface irradiated with Nd:YAG laser has not been studied yet. For these reasons, the aim of this *in vitro* study, is to evaluate the penetration of a bioceramic root canal sealer into dentinal tubules at 3 mm and 5 mm from the apex after Nd:YAG laser irradiation.

## Materials and methods

### Sample preparation

This study was approved by the Ethics Committee of University Nove de Julho (UNINOVE), São Paulo, Brazil (#1.358.755) (S1 File). Teeth gathered for this study came from patients who signed an informed consent document to grant their extracted teeth to the Human Teeth Biobank (HTB) of São Paulo University (S2 File). Forty mandibular uniradicular human premolars with completely formed root apexes and single canals confirmed by periapical radiography were used. Those teeth were chosen because it is easier to find mandibular premolars freshly extracted due to orthodontic reasons. Besides, they usually have circular root canals. Although maxillary central incisors often have a regular and circular root canal, it is a difficult tooth to find. Also, the diameter of maxillary central incisor root canal can be very large to prepare with Reciproc^®^ Files. The teeth were cleaned and stored at 4°C in a solution containing thymol grains for a maximum period of 3 months. The crowns of the teeth were sectioned transversally at the cementoenamel junction with a diamond disc (KG Sorensen, Barueri, SP, Brazil) and were the discarded. The exposed root canals were irrigated with 2 mL of 2.5% sodium hypochlorite, and a #10 K file (VDW GmbH, Munich, Germany) was introduced through the access cavity until it could be visualized at the apical foramen with a 2.5× magnifying glass. The instruments were removed and measured to determine the working length, which was set 1 mm from the acquired measure, which means 1mm from apical foramen. K file #​​20 or #25 instruments were used to determine the initial caliber. If one of these instruments did not fit, the teeth were discarded because the file system selected to prepare the root canals in this study were Reciproc^®^ R40 files (Reciproc^®^ System-VDW GmbH, Munich, Germany). The protocol of use recommended by the manufacturer, states that a manual file # 20 or # 25 must reach the working length passively to be prepared by a Reciproc^®^ R40 file. Subsequently, the teeth were prepared with R40 files, using the technique recommended by the manufacturer that consisted of 3 repeated penetrations and withdrawals until the working length was achieved. The teeth were irrigated with 2 mL of 2.5% in each cycle, with up to 10 mL in one cycle of the instrumentation. After this, the teeth were irrigated with 5 mL of 17% ethylenediaminetetraacetic acid (EDTA) and activated for 1 min with an Irrisonic^®^ ultrasonic tip (Helse Ultrassonic, Brazil) followed by irrigation with an additional 5 mL of 2.5% sodium hypochlorite. Subsequently, the teeth were randomly divided into 4 groups (n = 10): in group 1, the teeth were obturated with control sealer AH Plus^®^ sealer (Dentsply, Rio de Janeiro, Brazil); in group 2, they were obturated with bioceramic sealer Endosequence BC Sealer^®^ (Brasseler USA, Savannah, GA, USA); in group 3, they were irradiated with Nd:YAG laser + AH Plus^®^ sealer; and in group 4, they were irradiated with Nd:YAG laser + Endosequence BC Sealer^®^ (Brasseler USA, Savannah, GA, USA).

### Laser irradiation

The Nd:YAG laser (Deka, Florence, Italy), with a wavelength of 1064 nm and a 0.3-mm optical fiber, was used for irradiation. The standard parameters used were: 1.5 W, 15 Hz, and 100 mJ with a short pulse of 150 μs. The root canal wasn’t dried with paper cones. The root canal was left wet, only removing the sodium hypochlorite with a suction canula. After insertion of the optical fiber at the working length, the laser was activated, and the tooth was irradiated from the apical foramen to the entrance of the canal, with constant helical movements. Five cycles of 5 s were performed, with 20-s intervals between cycles. The irradiation protocol used in this study was the same as that proposed by Gutknecht & Behrens [40] and Camargo *et al*. [16], who recommended the use of helical movement to avoid excessive heating of the root canal walls.

### Root canal obturation

For all groups, bioceramic sealer Endosequence BC Sealer^®^ (Brasseler USA, Savannah, GA, USA) and AH Plus^®^ sealer (Dentsply, Rio de Janeiro, Brazil) were mixed with 0.1% rhodamine B (rhodamine B isothiocyanate; Sigma-Aldrich, St. Louis, MO, USA) prior to obturation. The root canal sealer was delivered in the root canal with a 1ml Luer tip syringe and an insertion tip provided by manufacturer of BC Sealer^®^, calibrated 2mm of working length. Teeth were obturated in a standard manner by the same operator using the R40 gutta-percha cone (VDW GmbH, Munich, Germany) and the vertical condensation technique. The samples were then placed in an incubator at 37°C under humidity (wet) for 1 week.

### Confocal laser scanning microscopy analysis

**Fig 1:**
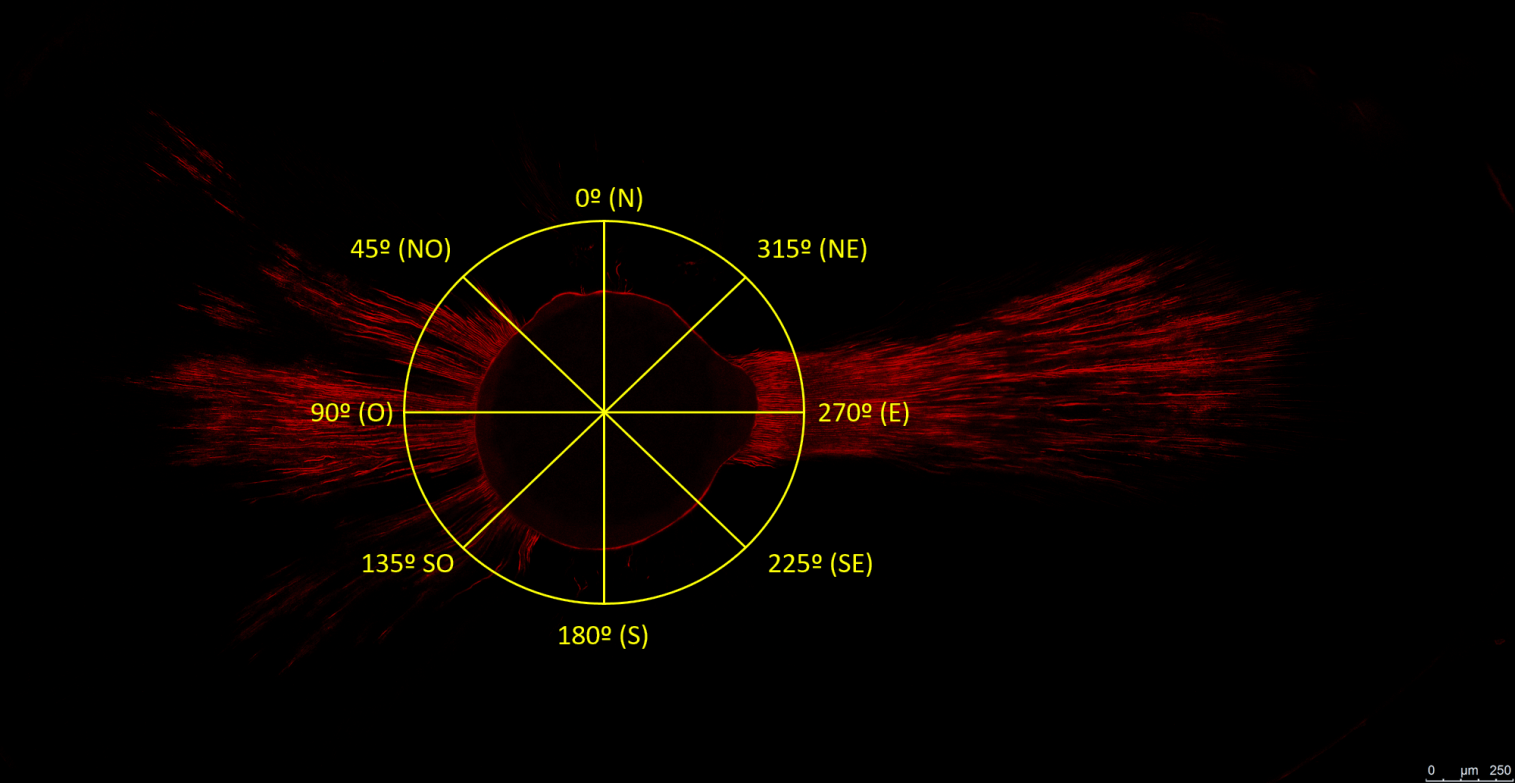
Reference octants for measuring the penetration depth of sealer.

After 1 week, the treated teeth were prepared for confocal laser scanning microscopy analysis. The teeth were cut perpendicular to the occlusal/apical axis at levels of 3 and 5 mm from the apex using a 0.3 mm #102 diamond disk mounted on an IsoMet Precision Cutter Saw (Buehler, Lake Bluff, IL) lubricated with vegetable oil. The samples were regularized with #600 and #1200 sandpaper for 10 s to reduce roughness and provide smooth surfaces, allowing intimate contact between the sample and glass slide during confocal microscopy. Subsequently, the specimens were examined using a confocal microscope (Leica TCS-SP 5 II, Leica, Mannheim, Germany) along the Z-axis with 10 μm optical slices. The absorption wavelength for rhodamine B was 543 nm. The displayed layers selected were 10 μm below the sample surface. For the analysis of penetration depth and the surface extension of sealer at each root level (i.e., 3 and 5 mm from the apex), 6 images containing the intracanal perimeter were captured for each sample with an HCX APO L 20×/ 1.0 lens (Leica) in oil (Type F Immersion Liquid) conforming to ISO 8036 (Leica), and acquired with the LAS AF Software (Leica Microsystems CMS GmbH, Mannheim). These images were obtained using 6 sections of 1 μm each, in a configuration of 1024 × 1024 pixels along the Z axis. Among these images, the one with the best sharpness was analyzed. The parameters analyzed were: penetration depth of the sealer in dentin tubules (in μm) and penetrated intracanal perimeter (in %) at a depth of 3 and 5 mm from the foraminal apex. In each micrograph selected, the maximum penetration depth of the sealer was measured in the 0° (N), 45° (NW), 90° (W), 135° (S), 180° (S), 225° (SE), 270° (E), and 315 ° (NE) directions (Fig 1) [21]. When penetration was observed, the length of the sealer tag formed in the canal wall along the tubule (in μm) was recorded. The canal wall served as the starting point and the penetration of the sealer into dentinal tubules (tags) was measured to a maximum depth of 1000 μm [41]. The intracanal perimeter of each sample was measured at a depth of 3 and 5 mm from the apex of the root. Both measurements (penetration and perimeter) were performed using an image-processing program (ImageJ, NIH, USA). The extension of the intracanal perimeter infiltrated by the sealer and the penetration percentages of the sealer were calculated. For statistical analysis, means of the measured variable were calculated for each sample and root canal section starting from the apex. (dx.doi.org/10.17504/protocols.io.rejd3cn)

### Statistical analysis

The data were analyzed for normality using the Kolmogorov-Smirnov test, and homogeneity using the Levene test. Two-way analysis of variance (ANOVA) with post hoc Bonferroni corrections was used for analyzing sealer penetration and sealer perimeter. The level of significance in all tests was 5%.

## Results

**Fig 2:**
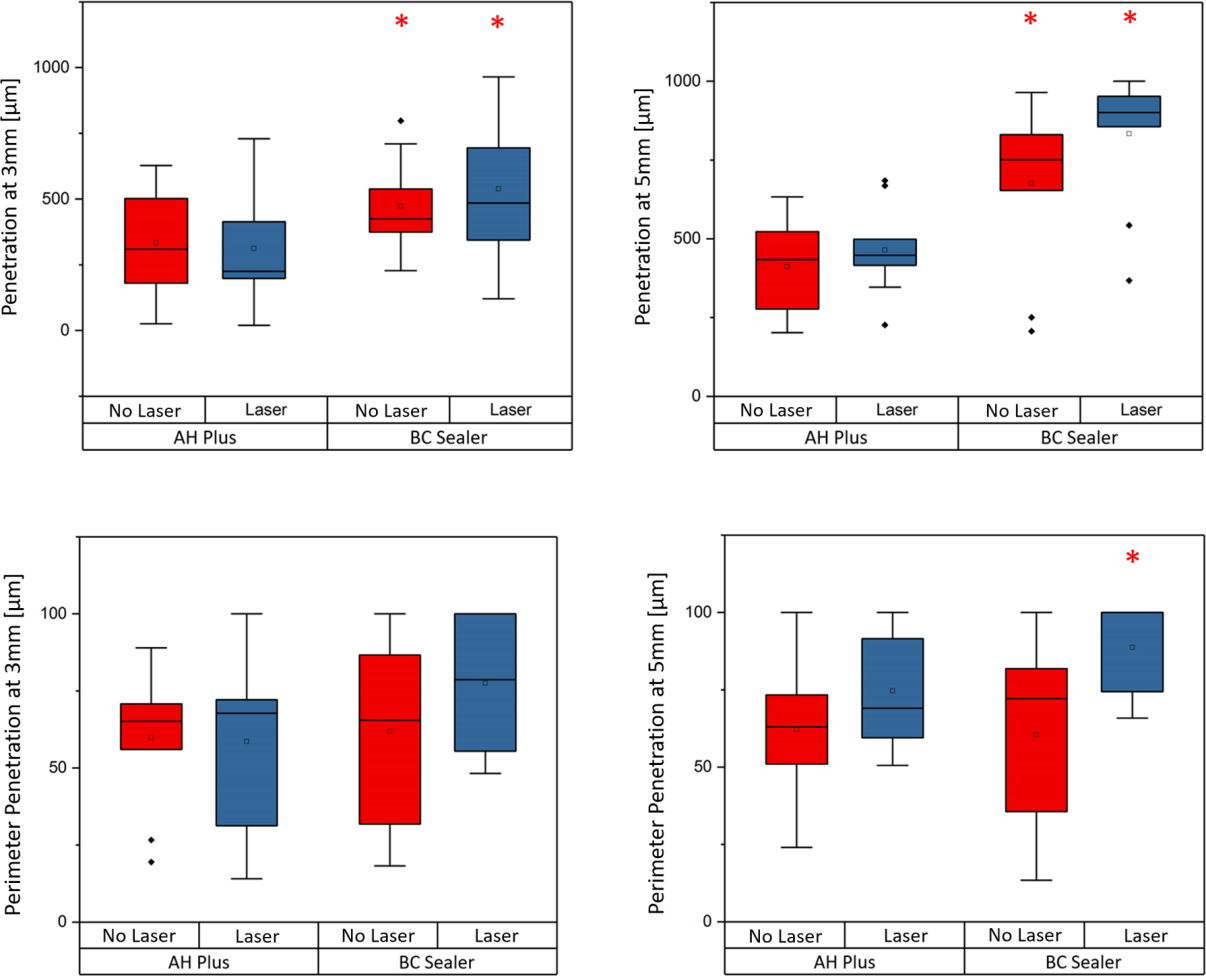
Results. Box plot graphics of measurements for sealer penetration at 3 and 5mm from apex and box plot graphics of measurements for perimeter of sealer penetration at 3 and 5mm from apex. *p<0,05.

**Fig 3:**
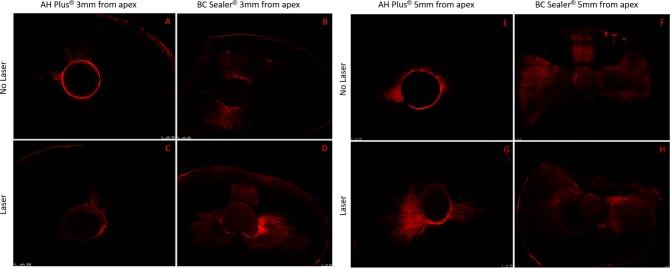
Representative images of the evaluated groups. (A) AH Plus^®^ 3mm from Apex and No Laser; (B) BC Sealer^®^ 3mm from Apex and No Laser; (C) AH Plus^®^ 3mm from Apex with Laser; (D) BC Sealer^®^ 3mm from Apex with Laser; (E) AH Plus^®^ 5mm from Apex and No Laser; (F) BC Sealer^®^ 5mm from Apex and No Laser; (G) AH Plus^®^ 5mm from Apex with Laser; (H) BC Sealer^®^ 5mm from Apex with Laser.

The results of this study are presented in Fig 2 and representative images of the evaluated groups are presented in Fig 3. When evaluating the sealer penetration at a depth of 3 mm from the apex (Fig 2), it was observed that bioceramic sealer (Endosequence BC Sealer^®^) showed a higher penetration (539 μm ± 257 μm) in the laser group (Fig 3D) and in the no laser group (473 μm ± 170 μm) (Fig 3B) than the control group (AH Plus^®^ sealer). In the control group, treatment with the laser resulted in a penetration of 312 μm (± 238 μm) (Fig 3C); and without the laser, the penetration at a depth of 3 mm was 333 μm (± 197 μm) (Fig 3A). Bioceramic sealer penetrated deeper into the dentinal tubules regardless of laser use at a depth of 3 mm from the apex (p <0.05); i.e., the laser did not prevent the penetration of sealer.

When evaluating the sealer penetration at a depth of 5 mm from the apex (Fig 2), the results were similar to that at a depth of 3 mm. Bioceramic sealer penetrated to 834 μm (± 210 μm) with the laser (Fig 3D) and 675 μm (± 249 μm) without the laser (Fig 3F), while the control sealer penetrated 464 μm (± 136 μm) with the laser (Fig 3G) and 415 μm (± 152 μm) without the laser (Fig 3E). This study also observed that bioceramic sealer penetrated deeper into the dentinal tubules regardless of laser use at a depth of 5 mm from the apex (p <0.05); i.e., the laser did not prevent the penetration of sealer.

For the penetrated intracanal perimeter (second outcome measure), we observed that there was no difference (p <0,05) between the bioceramic (59 μm ± 29 μm) and control group (77 μm ± 22 μm) at a depth of 3 mm from the apex for the laser groups (Fig 2). Additionally, without the laser, bioceramic sealer achieved a similar perimeter (62 μm ± 22 μm) as that of the laser group (60 μm ± 30 μm). At a depth of 5 mm from the apex (Fig 2), bioceramic sealer showed a larger perimeter penetration (89 μm ± 15 μm) with the use of the laser (p <0.05) than when the laser was not used (60 μm ± 30 μm).

## Discussion

This study evaluated the penetration of a bioceramic sealer in dentinal tubules irradiated by Nd:YAG laser, which was used as a coadjuvant for root canal decontamination. The objective was to combine the benefits of laser microbial reduction with the efficacy of bioceramic sealer [27,30,42–48]. Since Nd:YAG lasers can melt dentinal surfaces, this study examined whether this bioceramic sealer is capable of penetrating dentinal tubules despite laser irradiation. Penetration analysis showed that bioceramic sealer had a higher penetration at 3 and 5 mm than that of the control sealer, regardless of laser use. Thus, Nd:YAG laser does not interfere with the penetration of bioceramic sealer into dentinal tubules. Examination of the penetration perimeter showed that there was no difference between both sealers at a depth of 3 mm regardless of laser use. At a depth of 5 mm, bioceramic sealer paired with the laser showed a greater perimeter of penetration than that achieved with the control sealer, probably because of dentinal tubule size.

Assessment of sealer penetration at 3 mm and 5 mm from the apex in lower premolars demonstrated that bioceramic sealer had a higher penetration than control sealer, regardless of laser use. A possible explanation for this result is that bioceramic sealer has a higher flow than control sealer [29,43] with particles smaller than 2 μm [35] facilitating penetration into dentin tubules. Another explanation for the higher penetration of bioceramic sealer is its hydrophilicity, which improves penetration in moist substrates [43,45]. The control sealer (AH Plus^®^) contains epoxy resin that contributes to its hydrophobicity [49]. Based on the similarity between irradiated and non-irradiated groups with respect to dental sealer penetration, it can be assumed that lasers do not significantly altered dentin structure. This shows that the use of lasers is feasible as an auxiliary therapy without compromising root canal sealing.

We observed that there was no difference in the perimeter of sealer penetration between groups at a depth of 3 mm from the apex. This could be because of the smear layer, which is not completely removed by EDTA, especially in the apical region [50]. Additionally, the laser could have melted the residual smear layer, making sealer penetration more difficult. In this study, EDTA was applied in all groups to open the dentinal tubules. Another possibility could be the irregular distribution of the dentinal tubules at a depth of 3 mm from the apex. In buccal and lingual surfaces, the tubules are more parallel to the walls of the root canal [51,52], which may interfere with visualization using confocal microscopy. This would explain how the percentage of sealer penetration in the perimeter of the canal was close to 50% in the studied groups at a depth of 3 mm from the apex.

On the other hand, at a depth of 5 mm from the apex, we noticed a greater penetration (p <0.05) of sealer in the perimeter of the canal (close to 70% with the BC Sealer^®^) when paired with the use of laser. The greater penetration in the perimeter of the canal at a depth of 5 mm can be explained by the larger number and larger diameter of dentin tubules, as well as a more homogeneous distribution of the tubules [51,53]. However, there was no difference in perimeter penetration between the laser and no laser groups for the Endosequence BC Sealer^®^. It can be speculated that the conical shape created by the Reciproc^®^ instrument increases the area of ​​exposed dentinal tubules and allows both EDTA and the laser to be more efficient. Additionally, larger tubules may contain more humidity, which favors the penetration of bioceramic cement. Moreover, Endosequence BC Sealer^®^ expands 0.2% while it sets [35], allowing a greater contact between the sealer and root canal walls. This could explain why the laser group had a larger perimeter of penetrated bioceramic cement (89 ± 15% with the laser and 60 ± 30% without the laser), compared to control sealer. A limitation of this study was not to evaluate the sealer penetration at 2mm from the root apex [29]. Additional research could be necessary to evaluate sealer penetration in accessory canals.

Under the experimental conditions of this study, it can be concluded that the use of Nd:YAG laser did not compromise the penetration of bioceramic sealer into dentinal tubules of root canals at 3 mm and 5 mm from the apex. Further investigation is recommended.

## Supporting information

S1 File Ethics committee resolution. (PDF)

S2 File Declaration from Human Teeth Biobank. (PDF)
