# Supplementary material for: Influence of Nd:YAG laser on the penetration of a bioceramic root canal sealer into dentinal tubules: A confocal analysis
Source: PLoS One. 2018 Aug 22;13(8):e0202295. doi: 10.1371/journal.pone.0202295 (PMC6104986; doi:10.1371/journal.pone.0202295)
Supplement: S1 File — (PDF) [file pone.0202295.s001.pdf]

PARECER CONSUBSTANCIADO DO CEP

DADOS DO PROJETO DE PESQUISA

Título da Pesquisa: AÇÃO DE DIFERENTES PARÂMETROS DE LASER ND:YAG NA INTERAÇÃO ENTRE MATERIAIS OBTURADORES E A SUPERFÍCIE DENTINÁRIA DE DENTES TRATADOS ENDODONTICAMENTE.

Pesquisador: Rodrigo Jardim del Monaco

Área Temática:

Versão: 1

CAAE: 51537315.0.0000.5511

Instituição Proponente: ASSOCIACAO EDUCACIONAL NOVE DE JULHO

Patrocinador Principal: Financiamento Próprio

DADOS DO PARECER

Número do Parecer: 1.358.755

Apresentação do Projeto:

Os objetivos do preparo químico-cirúrgico do canal radicular são a limpeza, modelagem e desinfecção dos canais radiculares para posterior
selamento com a maior eficiência possível. Desta forma, o sucesso do tratamento endodôntico é dependente da técnica obturadora utilizada para o
selamento do canal radicular tratado. A interdependência entre a endodontia e o selamento dos canais radiculares é bem conhecida, entretanto, as
técnicas utilizadas para tal, são bastante variadas. O presente trabalho analisará a interação entre materiais obturadores e a superfície dentinária de
dentes tratados endodonticamente, após ação do laser Nd:YAG e EDTA 17% na limpeza da dentina radicular. Serão utilizados 68 dentes humanos
uniradiculares, os quais serão instrumentados com técnica reciprocante.
Um total de 68 dentes humanos será utilizado, sendo vinte dentes uniradiculares preparados com Reciproc® serão divididos em um grupo controle,
sem tratamento dentinário e 3 grupos onde serão ou não irrigados com EDTA 17% e irradiados ou não com laser Nd:YAG e serão avaliados quanto
ao maior número de túbulos dentinários abertos na superfície, através de MEV. Os mesmos

tratamentos dentinários serão realizados em outros 48
dentes uniradiculares. Estes serão divididos em grupos interagindo 2 diferentes cimentos obturadores, que serão avaliados por microscopia
confocal, totalizando 06 grupos. Os dados serão analisados de forma descritiva ou estatisticamente de acordo com as variáveis de resposta.

Objetivo da Pesquisa:

Objetivo geral:
Estudar a interação entre a dentina irradiada e o material obturador em raízes tratadas endodonticamente

Objetivo Secundário:

1-Avaliação morfológica com microscopia eletrônica de varredura (MEV) da superfície dentinária radicular frente à limpeza do canal radicular com ou
sem EDTA 17% e irradiação com laser Nd:YAG;
2-Análise morfológica com microscopia confocal da interface entre a dentina irradiada e diferentes materiais obturadores, utilizando os diferentes
tratamentos dentinários.

Avaliação dos Riscos e Benefícios:

Riscos:
O estudo não oferece riscos pois serão utilizados dentes oriundos do banco de dentes da Faculdade de Odontologia da Universidade de São Paulo.
Benefícios:
Os resultados desse estudo poderão ser aplicados na clínica odontológica melhorando a realização dos tratamentos endodônticos dos pacientes.

Comentários e Considerações sobre a Pesquisa:

A pesquisa é adequada

Considerações sobre os Termos de apresentação obrigatória:

Os termos estão adequados

Recomendações:

não ha recomendações

Conclusões ou Pendências e Lista de Inadequações:

Não ha inadequações

Considerações Finais a critério do CEP:

Este parecer foi elaborado baseado nos documentos abaixo relacionados:

Tipo Documento	Arquivo	Postagem	Autor	Situação
Informações Básicas do Projeto	PB_INFORMAÇÕES_BÁSICAS_DO_PROJETO_636450.pdf	01/12/2015 10:28:42		Aceito
Folha de Rosto	folhaderostoRJDMCONEP.docx	01/12/2015 10:27:54	Rodrigo Jardim del Monaco	Aceito
Cronograma	CRONOGRAMARJDMCONEP.docx	01/12/2015 10:20:45	Rodrigo Jardim del Monaco	Aceito
TCLE / Termos de Assentimento / Justificativa de Ausência	TERMOBIOBANCOFOUSP.pdf	01/12/2015 10:18:21	Rodrigo Jardim del Monaco	Aceito
Projeto Detalhado / Brochura Investigador	ProjetoRJDMCONEP.docx	01/12/2015 10:16:23	Rodrigo Jardim del Monaco	Aceito

Situação do Parecer:

Aprovado

Necessita Apreciação da CONEP:

Não

SAO PAULO, 09 de Dezembro de 2015

Assinado por:

Raquel Agnelli Mesquita Ferrari
(Coordenador)

Endereço: VERGUEIRO nº 235/249
Bairro: LIBERDADE **CEP:** 01.504-001
UF: SP **Município:** SAO PAULO
Telefone: (11)3385-9197 **E-mail:** comitedeetica@uninove.br
