# Supplementary material for: Influence of Nd:YAG laser on the penetration of a bioceramic root canal sealer into dentinal tubules: A confocal analysis
Source: PLoS One. 2018 Aug 22;13(8):e0202295. doi: 10.1371/journal.pone.0202295 (PMC6104986; doi:10.1371/journal.pone.0202295)
Supplement: S2 File — (PDF) [file pone.0202295.s002.pdf]

UNIVERSIDADE DE SÃO PAULO
Faculdade de Odontologia
Biobanco de Dentes Humanos
Nº do Registro na CONEP B-010

São Paulo, 11 de novembro de 2015.

Ao Comitê de Ética em Pesquisa:

O Biobanco de Dentes Humanos (divisão Permanentes) declara que possui estocados de acordo com as regras vigentes do CNS e poderá fornecer 70 (SETENTA) dentes caninos ou pré-molares unirradiculares inferiores humanos necessários ao projeto de pesquisa intitulado "AÇÃO DE DIFERENTES PARÂMETROS DE LASER ND:YAG NA INTERAÇÃO ENTRE MATERIAIS OBTURADORES E A SUPERFÍCIE DENTINÁRIA DE DENTES TRATADOS ENDODONTICAMENTE".

Pesquisador(a) responsável: Rodrigo Jardim Del Monaco

Orientador (a): Profa. Dra. Cristiane Miranda França

Obs.: Os dentes só serão liberados após vossa aprovação.

JCP Imparato

Prof. Dr. José Carlos P. Imparato
Responsável pelo Biobanco FOUSP
